# The Relationship between Carotid Atherosclerosis, Inflammatory Cytokines, and Oxidative Stress in Middle-Aged and Elderly Hemodialysis Patients

**DOI:** 10.1155/2013/835465

**Published:** 2013-09-25

**Authors:** Hongqi Ren, Xuan Zhou, Zhiyong Luan, Xiaomei Luo, Shujing Han, Qing Cai, Wang Rui, Yan Li

**Affiliations:** ^1^Department of Nephrology, Huaihai Hospital of Xuzhou Medical College, 226 Tongsan Road, Xuzhou 221004, China; ^2^Department of Special Diagnosis, Huaihai Hospital of Xuzhou Medical College, 226 Tongsan Road, Xuzhou 221004, China; ^3^Clinical Laboratory, Huaihai Hospital of Xuzhou Medical College, 226 Tongsan Road, Xuzhou 221004, China

## Abstract

*Objective*. To identify the relationship between microinflammation, oxidative stress, and carotid arterial stiffness in hemodialysis patients. *Methods*. The CAS **β** and PWV obtained by ultrasound technology were used to assess carotid arterial stiffness. We divided the patients into either the CAS group or the non-CAS group based on the presence or absence of CAS. The parameters of ALB, Ca, P, TC, HDL, LDL, TG, glucose, creatinine, and hs-CRP levels were routinely tested in both groups of patients. The levels of TNF-**α**, IL-6, and 8-isoprostane F2**α** were measured by ELISA. *Results*. A total of 42 patients were enrolled in the CAS group and 20 patients were enrolled in the non-CAS group. No significant differences between the CAS group and the non-CAS group were observed with respect to age, dialysis duration, DBP, BUN, Cr, TC, TG, HDL, LDL, and Hb. However, SBP , pulse pressure, and 8-isoprostane levels of the CAS group were higher than those of the non-CAS group. The hs-CRP, TNF-**α**, and IL-6 levels were elevated in both groups but showed no significant differences. *Conclusions*. Maintenance of hemodialysis patients exhibits a microinflammatory state that may lead to atherosclerosis. The roles of hypertension and oxidative stress may be more important.

## 1. Introduction

Atherosclerosis is an independent predictor of cardiovascular disease (CVD) related to patients with end-stage renal disease (ESRD). Numerous factors are involved in atherosclerosis for ESRD patients, some of which are reversible. Increasing evidence has demonstrated that atherosclerosis is a chronic microinflammatory disease [1–4].

Renal excretion dysfunction is common for maintenance with hemodialysis (MHD) patients. Toxins and cytokines accumulate in the body. The imbalance between the antioxygen free radical system and the oxygen free radical production system causes peroxidative damage and lipid peroxidative injury. Glycation end products and advanced oxidation protein products accumulate in the body, bind to specific monocyte-macrophage cell surface receptors, and stimulate vast numbers of adhesion molecules. Nuclear factor *κ*B (NF-*κ*B) is also stimulated resulting in increased secretion of inflammatory cytokines such as interleukin (IL) 1, IL-6, and tumor necrosis factor (TNF) *α* [5, 6]. Inflammatory cytokines and oxidative stress play an important role in dialysis-related cardiovascular events in MHD patients [7, 8].

In previous studies, systolic blood pressure (SBP), age, increased blood calcium (Ca) levels, and diabetes were demonstrated to be independent risk factors for carotid artery stiffness (CAS) in elderly hemodialysis patients [9, 10]. However, few studies have assessed the relationship between CAS, microinflammation, and oxidative stress in MHD patients. Thus, the present study is aimed to further investigate this relationship.

## 2. Materials and Methods

### 2.1. Study Subjects

Sixty-two stable hemodialysis patients were recruited from 2006 to February 2012. The enrolled patients met the following criteria: (1) age ≥ 45 years, (2) dialysis duration ≥ 3 months, (3) absence of cardiovascular events 3 months before the study, (4) absence of acute infection signs, (5) absence of severe malnutrition (serum albumin > 30 g/L), and (6) 4 hours of dialysis per session, 3 times each week, KT/V ≥ 1.2 [9, 10]. An informed consent was obtained from all of the patients and healthy control subjects enrolled in this study. This study was approved by the hospital ethics committee.

Among the enrolled patients, there were 43 males and 19 females, aged 45*∼*76 years (mean: 59.4 ± 7.6 years). The dialysis duration was 3–61 months (median: 34 months); the patients were divided into either the CAS or non-CAS group according to the presence or absence of CAS. A total of 42 patients were enrolled in the MHD + CAS group, including 31 males and 11 females, aged 45*∼*73 years (mean: 57.2 ± 7.8 years). The dialysis duration was 3–61 months (median: 34 months). The primary diseases included 32 cases of chronic glomerulonephritis, 6 cases of diabetic nephropathy, 2 cases of interstitial nephritis, and 2 cases of primary hypertension. A total of 20 patients were enrolled in the non-CAS group, including 12 males and 8 females, aged 47*∼*76 years (56.2 ± 8.7 years). The dialysis duration was 3*∼*56 months (median: 32 months). The primary diseases included 13 cases of chronic glomerulonephritis, 4 cases of diabetic nephropathy, and 3 cases of primary hypertension. For hemodialysis, a Fresenius 4008B hemodialysis machine, F6 polysulfone membrane dialyzer, and bicarbonate dialysate (138 mmoL/L sodium, 2.5 mmoL/L potassium, 1.5 mmoL/L Ca, 0.5 mmoL/L magnesium, and 32 mmoL/L bicarbonate) were used. The area of the dialyzer membrane was 1.3 m^2^, the dialysate flow rate was 500 mL/min, and the blood flow rate was 200–300 mL/min.

The patients who have hypertension received the antihypertension agents including amlodipine, benazepril, and metoprolol. All the patients received the phosphate binders and erythropoiesis-stimulating agents.

Healthy control subjects were selected from patients who received a physical examination in our examination center. There were 12 in total, including 7 males and 5 females, ages 45*∼*68 years (57.9 ± 7.8 years).

This study was approved by the Ethics Committee of our hospital, and all patients signed informed consent forms before being divided into groups.

## 3. Biochemical Assays

Three milliliters of fasting venous blood was collected from the dialysis patients (predialysis) and the healthy control subjects on the dialysis day and the physical examination day. After collection, the blood samples were sent to the clinical laboratory for analysis. A Hitachi 7600 automatic biochemical analyzer was used to detect serum albumin (ALB), Ca, phosphorus (P), total cholesterol (TC), high-density lipoprotein (HDL), low-density lipoprotein (LDL), triglycerides (TG), glucose, creatinine, and C-reactive protein (CRP) levels. 

### 3.1. Detection of Serum TNF-*α*, IL-6, and 8-Isoprostane F2*α* Concentrations

Seven milliliters of fasting venous blood was collected from the dialysis patients and the healthy control subjects on the dialysis day and the physical examination day. After 30 min, the samples were centrifuged at 3000 rpm/min for 15 min. The supernatant was divided into tubes and stored at −20°C for analysis. The samples were sent to be analyzed after all of them were collected. Before analysis the samples were placed in cold water or left at room temperature. The samples were then centrifuged at 3000 rpm/min for 15 min. The supernatant was collected for detection. The enzyme-linked immunosorbent assay (ELISA) method was applied to detect serum TNF-*α*, IL-6, and 8-isoprostane F2*α*. The ELISA kits for TNF-*α* and IL-6 were purchased from eBioscience. The kit for 8-isoprostane F2*α* was purchased from Cayman Chemical Company. The assays were performed according to the manufacturers' instructions.

### 3.2. Carotid Arterial Stiffness *β* (CAS*β*) and Pulse Wave Velocity (PWV) Determination

CAS*β* was detected to evaluate the carotid arterial stiffness. A Philips Philipus IU 22 Color Doppler Ultrasonic Diagnostic apparatus was used. One hour after dialysis, the high-resolution vascular probe (frequency: 5–13 MHz) was used to observe the carotid artery wall. In the long axis view, the M sampling line was adjusted to 2 cm below the carotid artery sinus, and at the outer membrane of the arteries were parallel to the artery wall. The ET function was initiated, images were stored after 3–5 consecutive stable waveforms were obtained, and the blood pressure readings were entered immediately during postprocessing. The instrument automatically calculated the stiffness of the side of carotid artery (*β*) and the pulse wave velocity (PWV). According to the literature, CAS *β* > 10 was defined as carotid arterial stiffness [11–13].

## 4. Statistical Methods

The experimental data was statistically analyzed using SPSS 15.0 software. The quantitative data was presented as mean ± standard deviation ($\overset{-}{x}\pm S$). The paired data was analyzed by *t*-test, and differences with *P* < 0.05 were considered significant.

## 5. Results

### 5.1. General Conditions

The age, dialysis duration, diastolic blood pressure (DBP), BUN, Cr, TC, TG, HDL, LDL, and Hb were not significantly different between the CAS and non-CAS groups. However, the SBP and pulse pressure (PP = SBP-DBP) values of the CAS group were higher than those of the non-CAS group (*P* < 0.05; Table 1). Both the CAS*β* and PWV values of the CAS group were higher than those of the non-CAS group and the normal control group (Table 2).

### 5.2. Serum TNF-*α*, IL-6, and 8-Isoprostane F2*α* Levels

The 8-isoprostane level was higher in the CAS group relative to that of the non-CAS group (68.77 ± 45.96 pg/mL versus 25.56 ± 27.48 pg/mL, *P* < 0.01). Compared to normal control group, the hs-CRP, TNF-*α*, and IL-6 levels were elevated in both the CAS and non-CAS groups; however, there were no statistical differences between the CAS and non-CAS groups (*P* > 0.05; Table 3).

## 6. Discussion

Under physiological conditions, the vessel diameter changes according to the changes in blood flow. With the increase in carotid arterial stiffness, the amplitude of the vessel change decreases in the systolic and diastolic phases. The vessel wall stiffness can be determined accurately by detecting the movement amplitude. Determination of CAS using the ultrasound Echotracking technique is a reliable method for evaluation of aortic stiffness with CAS*β* as a commonly used indicator [11–13]. Numerous factors affect the carotid arterial stiffness in ESRD patients. Previous studies reported that SBP, age, increased blood Ca, and diabetes were independent risk factors of CAS in elderly hemodialysis patients [9, 10]. However, few studies exist regarding the relationship between CAS, microinflammation, and oxidative stress in MHD patients. Therefore, we evaluated CAS using the ultrasound Echotracking technique. We also divided the MHD patients into the CAS and non-CAS groups to explore this relationship between CAS, microinflammation, and oxidative stress in MHD patients.

Toxin and cytokine excretion dysfunction is common in patients with chronic renal failure. The glycation end products and advanced oxidation protein products accumulate in the body, resulting in the microinflammation. In MHD patients, the progression of inflammation is aggravated by the high-capacity load, bioincompatibility of the dialysis membrane, microbial contamination of the dialysate, the presence and usage of the vascular access, and potential infections [5, 14–18]. The present study demonstrated that the levels of hsCRP, TNF-*α*, and IL-6 were higher than normal in both the CAS and non-CAS groups, indicating that a general state of microinflammation existed in the MHD patients. However, there were no significant differences between the CAS and non-CAS groups, indicating that inflammatory cytokines might be involved in the progression of atherosclerosis, although it is unlikely that they play a major role.

In MHD patients, the imbalance between the antioxygen free radical system and oxygen free radical production system causes peroxidative damage and lipid peroxidative injury, which are involved in the dialysis-related cardiovascular events in these patients [7, 8]. In recent years, 8-isoprostane F2*α* has been widely studied for the evaluation of oxidative damage. It is an unsaturated eicosane fatty acid located in the SN-2 position of the phospholipid. Hypoxia can activate phospholipase A2, leading to release of arachidonic acid (AA) from the cell membrane phospholipids. Phospholipase C may also be activated, leading to the AA release from inositol triphosphate. Through the noncyclooxygenase pathway and the oxygen free radical pathway, the oxygen free radicals act on cell membrane AA, generating 8-isoprostane F2*α* through *β* cleavage and recombination. The level of 8-isoprostane F2*α* reflects the level of lipid peroxidation specifically and accurately. Thus, the oxidative stress severity is an ideal biological indicator for the evaluation of oxidative stress and lipid peroxidation [19]. In the present study, we found that the 8-isoprostane F2*α* levels of the CAS group were higher than those of the non-CAS group, indicating that oxidative stress might play a more important role in the progression of atherosclerosis.

Most ESRD patients develop hypertension. A number of studies showed that the carotid arterial stiffness index and hypertension were closely related in ESRD patients [20, 21]. Our results also demonstrated that SBP, PP, and CAS*β* were closely related in elderly MHD patients. However, in CAS and Non-CAS groups, the SBP and PP were higher than those in the control; however, in CAS group the SBP and PP were higher than those of the non-CAS group. The cause that we think when CAS occurs, the PWV increases, the phase during which the reflection wave arrives at the central artery shifts to the systolic phase from the diastolic phase, and the systolic phase delay pressure wave can be observed, leading to elevated SBP, decreased DBP, and increased pulse pressure. This increased pressure can cause arterial endothelial dysfunction leading to alterations of the systolic and diastolic functions of the arterial wall [19] and proliferation and fibrosis of the smooth muscle cells in the intercellular layer of the artery. Excessive collagen synthesis can be stimulated, resulting in arterial wall thickening and increased stiffness. Thus, hypertension and arteriosclerosis have causal interactions.

In conclusion, the present study demonstrated that the CRP, TNF-*α*, MCP-1, and IL-6 levels were higher than normal in both the CAS and non-CAS groups, indicating that there was a microinflammatory state in the MHD patients. The SBP and 8-isoprostane F2*α* levels were higher in the CAS patients relative to those of the non-CAS patients, indicating that atherosclerosis is more strongly related to SBP and oxidative stress.

## Figures and Tables

**Table 1:** Basic conditions of the study groups.

	Control	Non-CAS	CAS
Age (years)	57.9 ± 7.8	56.6 ± 9.6	57.2 ± 7.8

Gender (M/F)	7/5	12/8	31/11
Duration of dialysis (months)	/	32 (3–56)	34 (3–61)
SBP (mmHg)	131.08 ± 7.66	137.27 ± 11.23*	151.33 ± 13.57^∗∗▲^
DBP (mmHg)	72.66 ± 9.62	83.25 ± 10.48*	81.62 ± 11.35*
Pulse pressure (mmHg)	47.21 ± 8.37	56.96 ± 6.18	67.26 ± 18.22^∗∗▲^
BUN (mmol/L)	5.13 ± 0.69	23.36 ± 10.34**	23.31 ± 8.49**
Scr (*μ*mol/L)	62.58 ± 13.29	756.6 ± 131.8**	862.1 ± 157.4**
Hb (g/L)	14.33 ± 1.30	86.4 ± 24.6**	80.3 ± 25.5**
TC (mmol/L)	4.22 ± 0.61	4.26 ± 1.38	4.32 ± 1.22
TG (mmol/L)	1.74 ± 0.29	1.27 ± 0.37	1.32 ± 0.84
HDL-c (mmol/L)	1.37 ± 0.26	1.34 ± 0.23	1.27 ± 0.33
LDL-c (mmol/L)	2.49 ± 0.73	2.52 ± 0.84	2.43 ± 0.61

*P < 0.05 versus control; **P < 0.01 versus control; ^▲^ *P* < 0.05 versus non-CAS.

**Table 2:** Comparisons of CAS indicators in the study groups.

	Control	Non-CAS	CAS
*β*	7.18 ± 2.12	7.38 ± 2.84	12.14 ± 6.21^∗∗▲^
PWV	5.93 ± 0.52	6.26 ± 0.62	7.52 ± 0.51^∗∗▲^

**P < 0.01 versus control; ^▲^ *P* < 0.05 versus non-CAS.

**Table 3:** Comparisons of serum TNF-*α*, IL-6, and 8-isoprostane F2*α* concentrations in the study groups.

	Control	Non-CAS	CAS
TNF-*α* (pg/mL)	34.83 ± 10.46	83.67 ± 23.626*	79.4 ± 21.57*
IL-6 (pg/mL)	42.18 ± 13.47	128.36 ± 33.24*	119.56 ± 25.62*
hs-CRP (mg/L)	1.58 ± 0.79	7.54 ± 6.51*	8.21 ± 7.63*
8-isoprostane F2*α* (pg/mL)	17.32 ± 7.47	25.56 ± 27.48*	68.77 ± 45.96**

*P < 0.05, non-CAS versus control and CAS versus control; **P < 0.01, non-CAS versus CAS.
